# Comparative Evaluation of USG-Guided Single Tissue Marker Versus Multiple Tissue Marker Placements in Breast Malignancy Patients Undergoing Neoadjuvant Chemotherapy for Tumor Localization

**DOI:** 10.7759/cureus.65355

**Published:** 2024-07-25

**Authors:** Yashaswinii P, Evangeline P Christina, Sukumar Ramaswami, Harish Sl, Paarthipan Natarajan

**Affiliations:** 1 Radiodiagnosis, Saveetha Medical College and Hospitals, Saveetha Institute of Medical and Technical Sciences, Saveetha University, Chennai, IND

**Keywords:** usg-guided procedures, neoadjuvant chemotherapy, clip migration, breast carcinoma, tissue marker

## Abstract

Background

Breast cancer remains one of the most common malignancies affecting women globally, contributing significantly to the disease burden. The advent of neoadjuvant chemotherapy (NAC) has revolutionized the treatment for locally advanced breast cancer, allowing tumors to be downstaged and making breast-conserving surgery (BCS) feasible. Accurate localization of the tumor bed post-NAC is crucial for successful surgical removal of residual disease. While traditional single tissue marker placement has been effective, recent advances suggest multiple markers might provide superior localization by comprehensively delineating the entire tumor area. This study aims to compare the effectiveness of single versus multiple tissue marker placements in breast malignancy patients undergoing NAC.

Materials and methods

A prospective study was conducted in the Department of Radio-diagnosis at Saveetha Medical College over 18 months, including 10 patients diagnosed with breast carcinoma, selected through convenience sampling. Inclusion criteria involved patients diagnosed with breast cancer via mammography, sonography, and histological confirmation, referred for clip placement before NAC. Exclusion criteria were patients unwilling to participate. The procedure involved placing one to two surgical clips within the tumor using a 14/16-gauge coaxial guiding needle under USG guidance, with additional clips for larger or multiple tumors. Data collection included pre-procedural USG, post-procedural mammography (MG1), pre-operative mammography (MG2)/USG, and gross specimen histopathological examination/specimen mammography.

Statistical analysis

Demographic data, clipping distribution, receptor status, localization methods, surgical outcomes, operation diagnoses, and correlation analysis were statistically analyzed. Mean age, standard deviation, and p-values were calculated to determine the significance of differences between single and multiple clip groups.

Results

The study included 10 patients with a mean age of 52.5 years. Of these, five (50%) had a single clip, and two (20%) had four clips. The average time from clipping to the second mammogram (MG2) was 106.3 days, and from clipping to operation was 111.0 days, with longer follow-up times for multiple clip patients.

Six (60%) of the patients were estrogen receptor (ER) positive, and six (60%) were human epidermal growth factor receptor 2 (HER2) negative. Localization methods were similar between single and multiple clip groups. However, multiple clip patients tended to undergo more extensive surgeries like modified radical mastectomy (MRM). Imaging responses showed no preoperative ultrasound lesions in single clip patients, while multiple clip patients had higher inconsistent diagnoses (10 (100%)) suggesting that multiple clips provide better tumor localization but are linked to increased complexity and longer follow-up times.

Conclusion

Patients with multiple clips experienced significantly longer follow-up times, reflecting more complex clinical scenarios. Despite no significant differences in receptor status distributions, multiple clip patients required more extensive surgeries, emphasizing the need for tailored surgical planning. The study underscores the importance of considering the number of clips in clinical decision-making. Future research should focus on larger, prospective studies to validate these findings and explore underlying mechanisms.

## Introduction

Breast cancer remains one of the most common malignancies affecting women globally, accounting for a significant burden of disease. According to the World Health Organization (WHO), breast cancer is the most frequently diagnosed cancer among women, with over two million new cases in 2020 alone [1]. The advent of neoadjuvant chemotherapy (NAC) has revolutionized the treatment paradigm for locally advanced breast cancer. NAC can downstage tumors, making them amenable to breast-conserving surgery (BCS) rather than mastectomy, and allows for the assessment of tumor response to therapy [2].

Accurate localization of the tumor bed post-NAC is crucial for the successful surgical removal of residual disease. This is especially important in cases where NAC results in significant tumor shrinkage or complete pathological response. Tissue marker placement, guided by USG, is a well-established method to mark the tumor site before NAC, aiding surgeons in identifying the original tumor location during surgery [3]. Traditionally, a single tissue marker is placed within the tumor, but recent advances suggest that multiple markers might provide superior localization by delineating the entire tumor area more comprehensively [4].

The precision of tumor localization in breast cancer surgery is paramount to achieving clear surgical margins and minimizing the need for re-excision, which can affect cosmetic outcomes and increase patient morbidity [5]. Single tissue marker placement, while effective, may not adequately mark the entire tumor extent, particularly after a significant response to NAC. Multiple tissue marker placements could theoretically enhance localization accuracy by providing multiple reference points around the tumor periphery, thereby aiding in more precise surgical excision [6].

Despite the potential advantages, there is limited research comparing the effectiveness of single versus multiple tissue marker placements in this context. Understanding the relative benefits and limitations of each approach could inform clinical practice and improve surgical outcomes for breast cancer patients undergoing NAC.

This study aims to address this knowledge gap by conducting a comparative evaluation of these two localization techniques. This study has significant clinical implications as it addresses a critical component of breast cancer surgery, accurate tumor localization post-NAC. By comparing single and multiple tissue marker placements, the research aims to provide evidence-based recommendations that could enhance surgical precision and patient outcomes. Improved localization techniques could lead to better surgical margins, reduced re-excision rates, and ultimately, better overall prognosis for breast cancer patients [7]. The findings of this study could influence surgical protocols and contribute to the refinement of breast cancer treatment strategies.

## Materials and methods

This prospective study was conducted in the Department of Radio-diagnosis at Saveetha Medical College over a duration of 18 months. The study commenced after obtaining approval from the Institutional Human Ethics Committee (IHEC) of Saveetha Medical College (ECR/724/Inst/TN /2015/RR-19) and informed and written consent from all participants. The sample size consisted of 10 patients, selected through convenience sampling. The study population included all patients diagnosed with breast carcinoma, presenting to the hospital for treatment.

**Figure 1:**
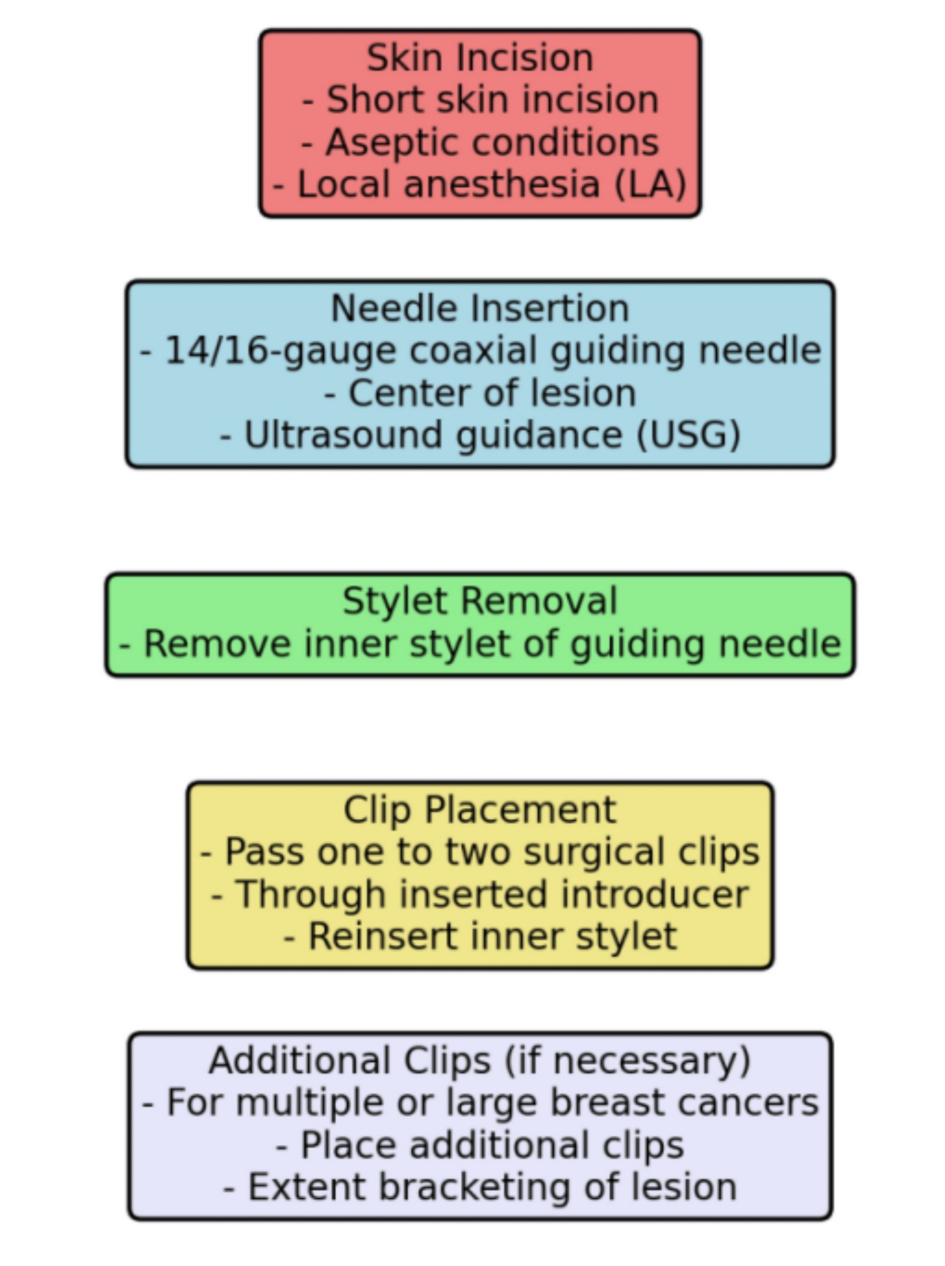
Pathway and procedure of clip insertion

Patients were included in the study if they had an initial diagnosis of breast cancer confirmed by mammography, sonography, and subsequent histological confirmation. Clinical staging, including palpable tumor size and lymph node status, was recorded for each patient. Prior to starting NAC, patients were referred to the Department of Radio-diagnosis for the placement of tissue markers. Patients who were not willing to participate in the study were excluded. The procedure for clip placement is shown in Figure 1.

Involved making a short skin incision under local anesthesia and aseptic conditions. A 14/16-gauge coaxial guiding needle was inserted into the center of the lesion, with the inner stylet removed under USG guidance. One to two surgical clips were then passed through the inserted introducer, and the inner stylet was reinserted to complete the clip placement. Additional clips were placed for larger or multiple tumors to bracket the lesion. The location of the clips was confirmed by USG immediately after insertion, with the coaxial needle appearing as an echogenic white line and the clips as linear hyperechoic structures with or without posterior acoustic shadowing.

**Figure 2:**
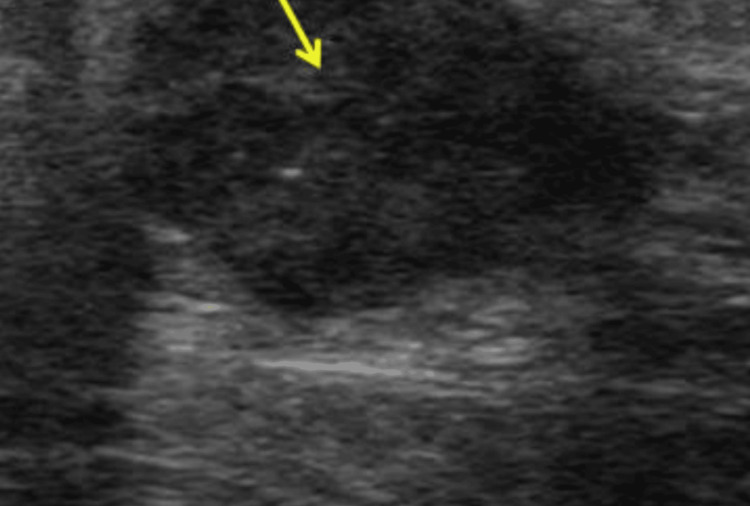
Initial ultrasound of left breast findings Ill-defined hypoechoic lesion (yellow arrow) with radiating borders and adjacent ductal dilatation reaching upto the nipple of size ~2.8x2 cm in 3 o’clock position within zone 2b-BIRADS 4.

Pre-procedural USG-Ill defined hypoechoic lesion with internal vascularity, radiating borders, and adjacent ductal dilatation reaching upto the nipple of size ~2.8x2 cm in 3 o’clock position within zone 2b-BIRADS 4 (Figure 2).

**Figure 3:**
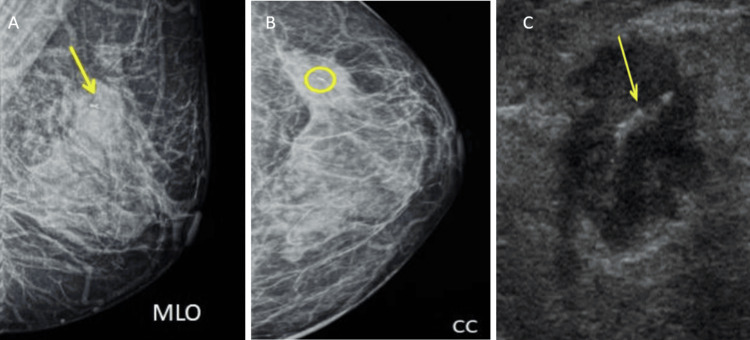
MG1: A) MLO, B) CC, and C) post-procedural USG image A) Opacity with partly defined borders retro-mammary tenting and architectural distortion in the upper quadrant of the left breast (yellow arrow), B) radio-opaque ribbon-shaped marker (yellow circle) seen within the above-mentioned lesion, and C) a linear hyperechoic structure - clip (yellow arrow) within the center of the lesion. In this case, only a single marker was placed. MLO, mediolateral oblique view; CC, craniocaudal view; MG1, post-procedural mammography

Post-procedural mammography (MG1) and USG show opacity with partly defined borders retro-mammary tenting and architectural distortion in the upper quadrant of the left breast, BIRADS 6 (Figures 3A, 3B). USG image shows a linear hyperechoic structure, clip (yellow arrow) within the center of the lesion. In this case, only a single marker was placed (Figure 3C).

**Figure 4:**
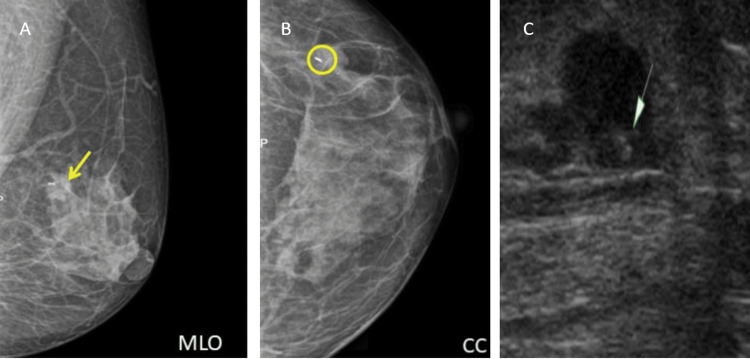
MG2: A) MLO, B) CC view, and C) post-chemotherapy/pre-operational USG A) MLO view - there is a significant reduction of the tumor (yellow arrow) in comparison to the initial mammography MLO, B) CC view - radio-opaque ribbon-shaped marker (yellow circle) seen at the site of the assumed lesion and there is no evidence of clip migration, and C) USG - a linear hyperechoic structure - clip (green arrow) within the center of the significantly reduced lesion. MLO, mediolateral oblique view; CC, craniocaudal view; MG2, post-chemotherapy/pre-operational mammography

Post-chemotherapy/pre-operational mammography (MG2) findings show that there is a significant reduction of the tumor (yellow arrow) in comparison to the initial mammography and radio-opaque ribbon-shaped marker (yellow circle) seen at the site of the assumed lesion and there is no evidence of clip migration (Figures 4A, 4B); and post-chemotherapy/pre-operational USG findings: USG image shows a linear hyperechoic structure - clip (green arrow) within the center of the significantly reduced lesion (Figure 4C).

**Figure 5:**
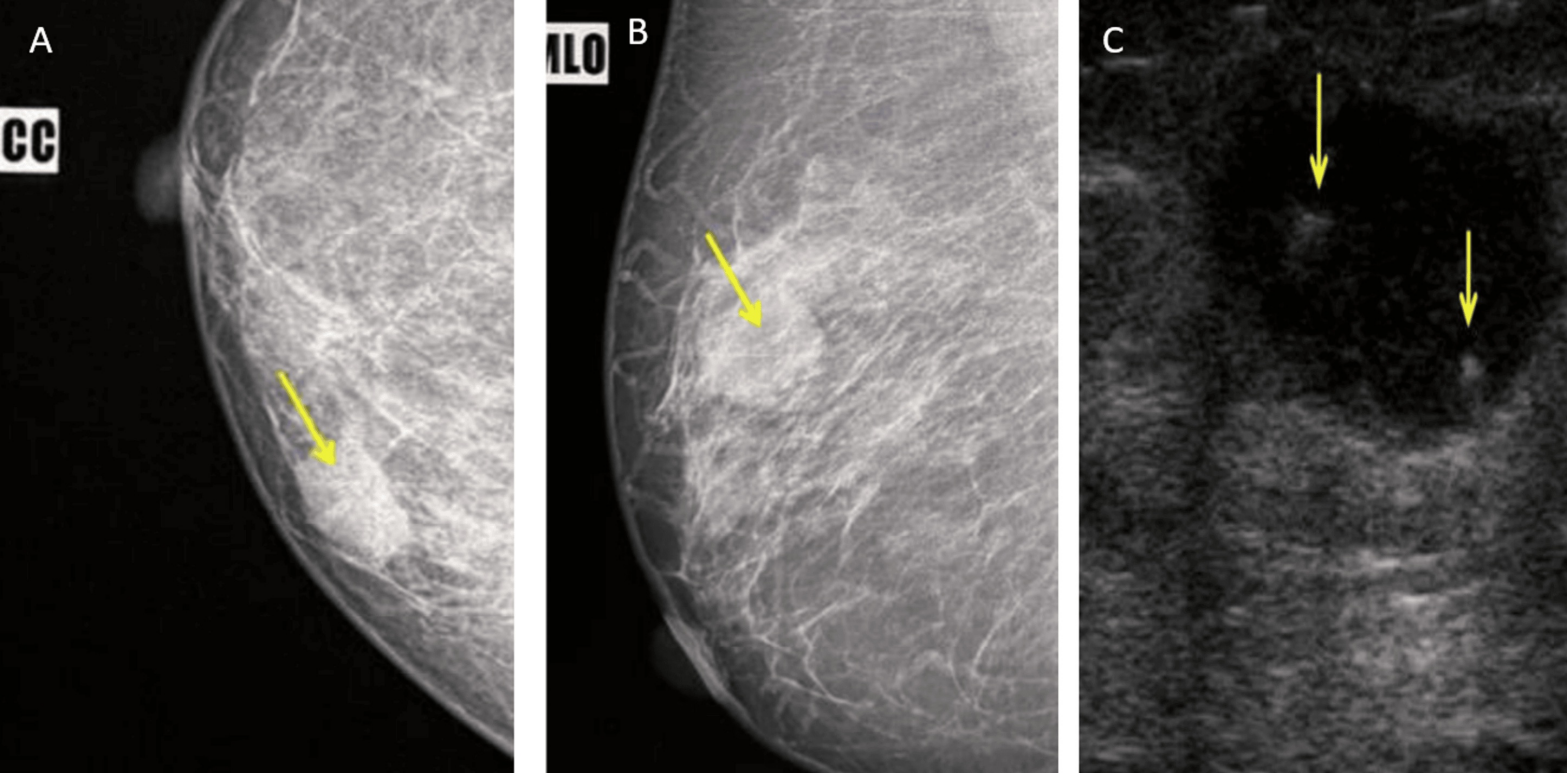
Pre-procedural mammography: A) CC, B) MLO, and C) post-procedural USG A) Cranio-caudal view opacity with partly defined borders and mild architectural distortion in the upper inner quadrant of the right breast - BIRADS 4 (yellow arrow). B) MLO view shows the same lesion (yellow arrow). C) Two linear hyperechoic structures - clip (yellow arrows) within the center of the lesion. In this case, four markers were placed. MLO, mediolateral oblique view; CC, craniocaudal view

Pre-procedural mammography (MG2) findings: A) cranio-caudal view shows opacity with partly defined borders and mild architectural distortion in the upper inner quadrant of right breast - BIRADS 4 and MLO view shows the same lesion (yellow arrow) (Figures 5A, 5B). The corresponding USG image shows two linear hyperechoic structures - clip (yellow arrows) within the center of the lesion (Figure 5C). 

**Figure 6:**
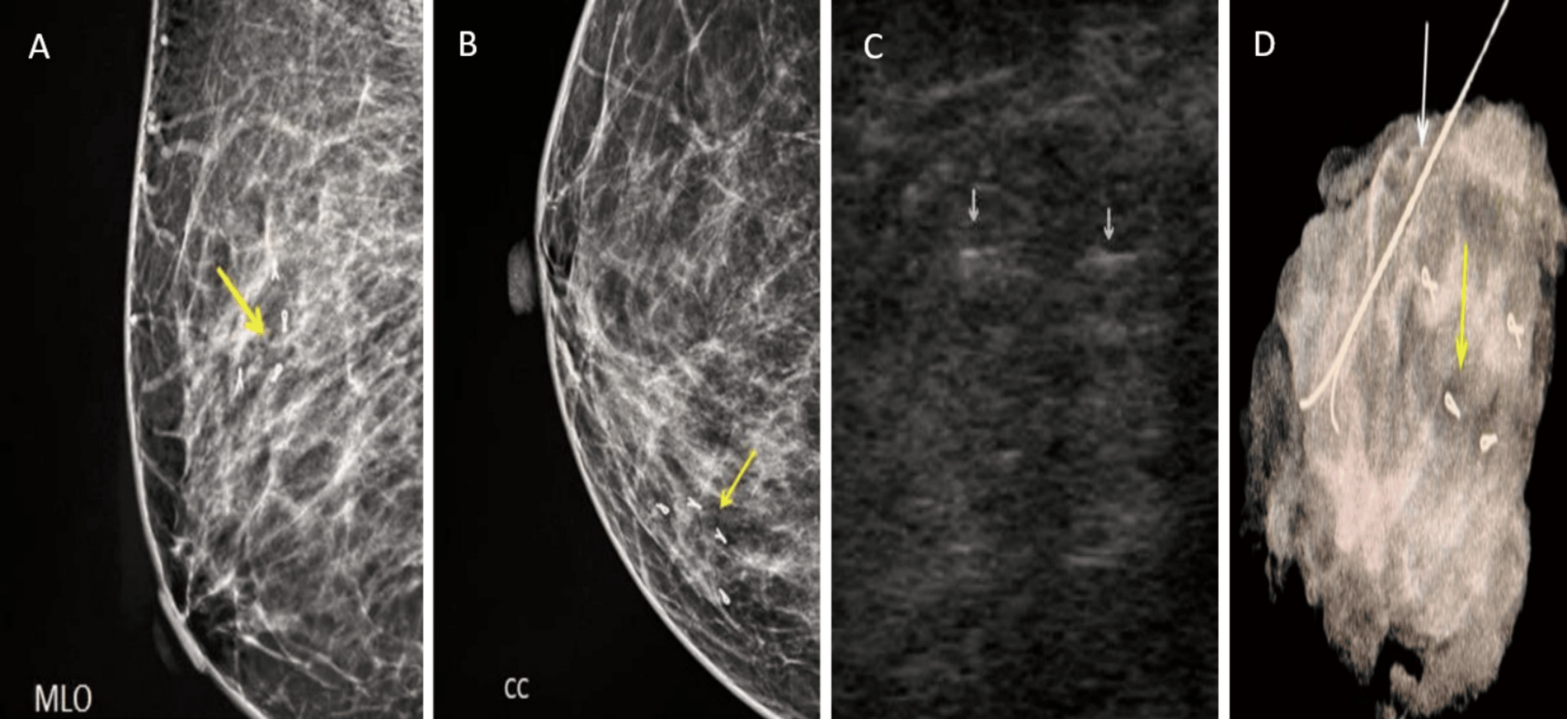
MG2: A) MLO, B) CC, C) post-chemotherapy/pre-operational USG, and D) specimen mammography A) MLO view shows there is significant reduction of the tumor (yellow arrow) in comparison to the initial mammography. B) CC view shows four radio-opaque ribbon-shaped markers seen at the site of assumed lesion (yellow arrow) and there is no evidence of clip migration. Post-chemotherapy/pre-operational USG findings: C) USG image shows two linear hyperechoic structures - clips (white arrows) at the assumed site of the lesion. There is significant reduction in the size of the lesion. D) Specimen mammography findings show a linear wire structure (white arrow) within the excised lesion, and all four clip markers (yellow arrow) were removed and were seen within the lesion with no evidence of clip migration. MLO, mediolateral oblique view; CC, craniocaudal view; MG2, post-chemotherapy/pre-operational mammography

Post-chemotherapy/pre-operative mammography (MG2) findings indicate a significant tumor reduction in the MLO view and the presence of four radio-opaque ribbon-shaped markers at the lesion site in the CC view, with no clip migration (Figures 6A, 6B). USG shows two linear hyperechoic structures (clips) at the lesion site, with a significant reduction in lesion size (Figure 6C). Specimen mammography reveals a linear wire structure within the excised lesion, with all four clip markers present within the lesion and no evidence of clip migration (Figure 6D).

## Results

**Figure 7:**
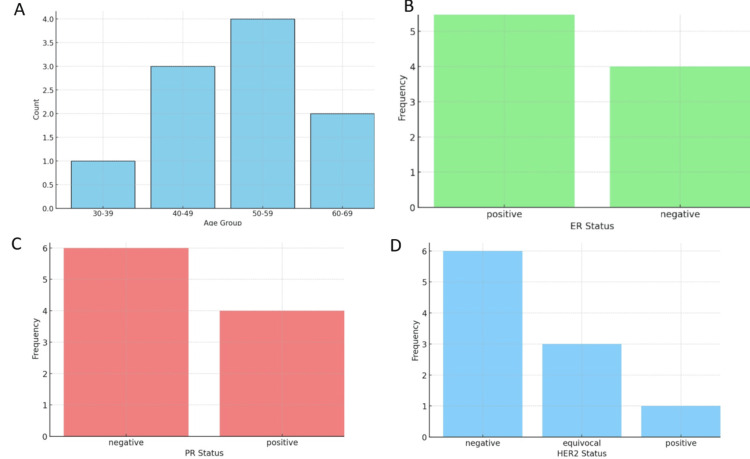
A) Age distribution, B) ER distribution, C) PR distribution, and D) HER2 receptor distribution HER2, human epidermal growth factor receptor 2; ER, estrogen receptor; PR, progesterone receptor

The age distribution of the patients reveals that 10 (10%) are in the 30-39 age group, three (30%) in the 40-49 age group, four (40%) in the 50-59 age group, and two (20%) in the 60-69 age group (Figure 7A). 

The ER status distribution shows that six (60%) of the patients were ER-positive, and four (40%) were ER-negative, indicating a majority of the patients had ER-positive tumors (Figure 7B).

The progesterone receptor (PR) status distribution reveals that four (40%) of the patients were PR-positive, while six (60%) were PR-negative, indicating a higher prevalence of PR-negative tumors among the patients (Figure 7C).

The human epidermal growth factor receptor 2 (HER2) status shows that six (60%) of the patients were HER2 negative, three (30%) were equivocal, and 10 (10%) were HER2 positive, indicating that most patients had HER2-negative status, with a significant portion having ambiguous results (Figure 7D).

Regarding clipping distribution, five (50%) of patients had one clip, two (20%) had two clips, three (30%) had three clips, and two (20%) had four clips. This indicates that most patients had a single clip, with fewer requiring multiple clips (Table 1). 

**Table 1:** Clipping distribution

Number of clips	N (%)
1	5 (50%)
2	2 (20% )
3	3 (30% )
4	2 (20% )

The average time from clipping to the second mammogram (MG2) was 106.3 days with a standard deviation of 20.19 days, and from clipping to operation was 111.0 days with a standard deviation of 19.68 days. The range for clipping to MG2 was 75 to 132 days, while for clipping to operation, it was 78 to 140 days, indicating a similar time frame for both follow-up activities (Table 2).

**Table 2:** Time from clipping to mammogram and operation MG2, post-chemotherapy/pre-operational mammography

Metric	Clipping to MG2 and USG	Clipping to operation
Mean	106.3 days	111.0 days
Standard deviation	20.19 days	19.68 days
Range	75 to 132 days	78 to 140 days

Patients with multiple clips had a mean time of 120.4 days (SD: 11.52 days) from clipping to MG2 and USG, while those with a single clip had a mean time of 92.2 days (SD: 16.95 days). The difference is statistically significant with a p-value of 0.0057, indicating longer times for patients with multiple clips. The mean time from clipping to operation was 124.2 days (SD: 13.61 days) for patients with multiple clips and 97.8 days (SD: 15.83 days) for those with a single clip. This difference is also statistically significant with a p-value of 0.0046, showing longer wait times for patients with multiple clips (Table 3).

**Table 3:** Comparative analysis: single versus multiple clips - clipping to MG2 and USG and clipping to operation *Represents a significant p-value. MG2, post-chemotherapy/pre-operational mammography

Clip type	Mean time (days)	Standard deviation (days)	p-value
Clipping to MG2 and USG (single clip)	92.2	16.95	0.0057*
Clipping to MG2 and USG (multiple clips)	120.4	11.52	0.0057*
Clipping to MG2 and USG (single clip)	97.8	15.83	0.0046
Clipping to operation (Multiple clips)	124.2	13.61	0.0046

Among patients with multiple clips, there was no significant difference in ER status (p=0.3633), PR status (p=0.2405), or HER2 status (p=0.5714) compared to single clip patients. ER status distribution: multiple clips (4 ER+ (80%), 1 ER- (20%)), single clip (2 ER+ (40%), 3 ER- (60%)). PR status distribution: multiple clips (3 PR+ (60%), 2 PR- (40%)), single clip (1 PR+ (20%), 4 PR- (80%)). HER2 status distribution: multiple clips (two equivocal (40%), two negative (40%), one positive (20%)), single clip (one equivocal (20%), four negative (80%), 0 positive (0%)) (Table 4).

**Table 4:** Comparison of ER, PR, and HER2 status between single versus multiple clips ER, estrogen receptor; PR, progesterone receptor; HER2, human epidermal growth factor receptor 2

Receptor status	Single clip, N (%)	Multiple clips, N (%)	p-value
ER positive	2 (40%)	4 (80%)	0.3633
ER negative	3 (60%)	1 (20%)	0.3633
PR positive	1 (20%)	3 (60%)	0.2405
PR negative	4 (80%)	2 (40%)	0.2405
HER2 equivocal	1 (20%)	2 (40%)	0.5714
HER2 negative	4 (80%)	2 (40%)	0.5714
HER2 positive	0 (0%)	1 (20%)	0.5714

All patients with single clips (5, 100%) underwent breast conservation surgery (BCS), whereas two (40%) of multiple clip patients had BCS and three (60%) had modified radical mastectomy (MRM); the p-value was 0.086, indicating no significant difference. Diagnoses for multiple clip patients included 40% (N=2) ductal carcinoma in situ (DCIS), 20% (N=1) invasive ductal carcinoma (IDC), and 2 (40%) invasive carcinoma of no special type (NST), while single clip patients had three (60%) IDC and 2 (40%) NST, with no DCIS cases. Comparative analysis showed two DCIS cases (40%) in multiple clips, none in single clips, one IDC case (20%) in multiple clips, three (60%) in single clips, and two NST cases (40%) in both groups, with a p-value of 0.262 indicating no significant difference (Table 5).

**Table 5:** Frequency of operation diagnoses for single and multiple clips and comparison of frequency of operation diagnoses for multiple and single clips BCS, breast conservation surgery; MRM, modified radical mastectomy; DCIS, ductal carcinoma in situ; ICS, invasive ductal carcinoma; NST, invasive carcinoma of no special type

Surgery type/diagnosis type	Single clip, N (%)	Multiple clips, N (%)	p-value
BCS	5 (100%)	2 (40%)	0.086
MRM	0 (0%)	3 (60%)	0.086
DCIS	0 (0%)	2 (40%)	0.262
IDC	3 (60%)	1 (20%)	0.262
NST	2 (40%)	2 (40%)	0.262

**Figure 8:**
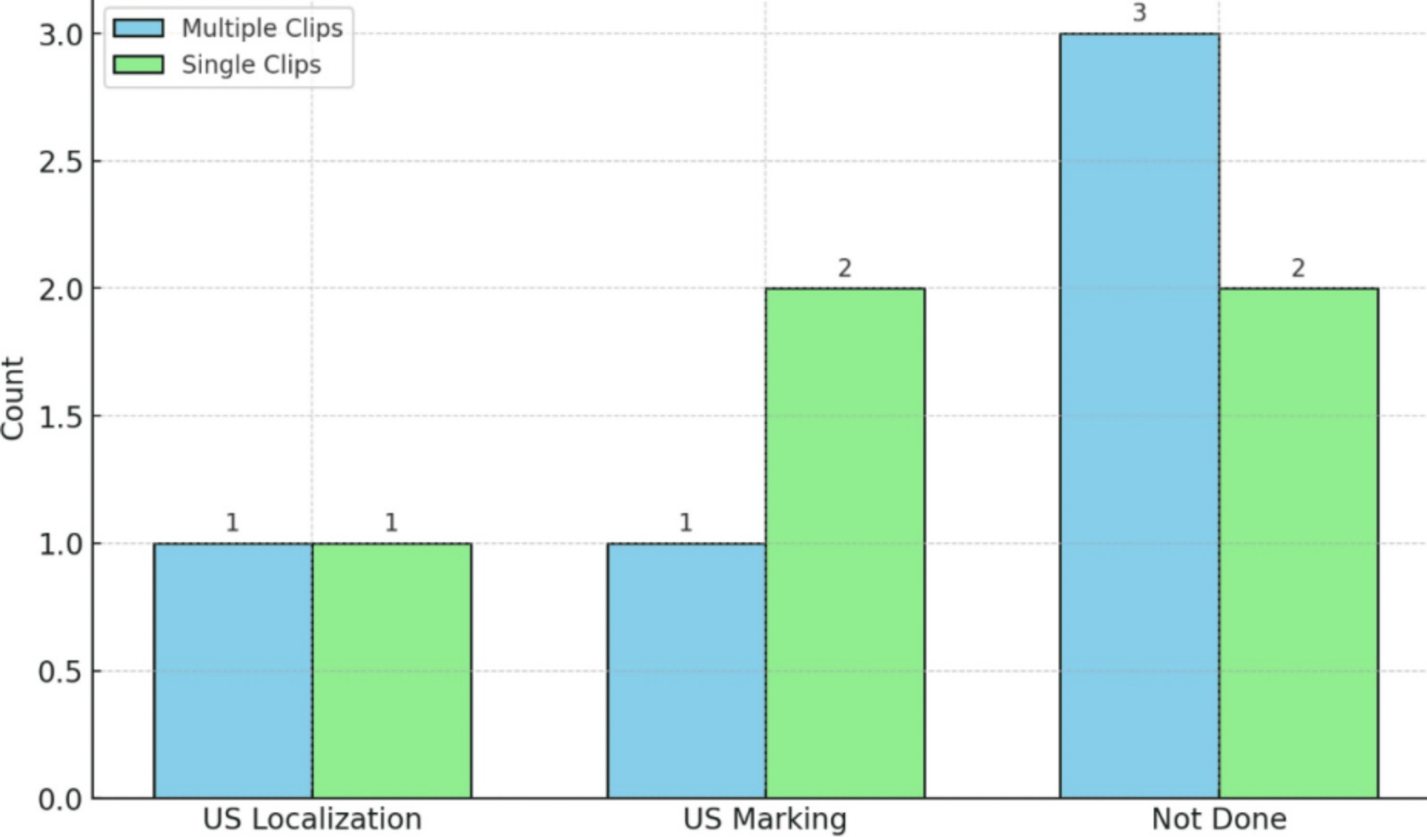
Comparison of frequency of localization methods for multiple and single clips

The comparison shows that one patient each in both multiple (20%) and single clip groups (20%) used US localization, one in the multiple clips group (20%) and two in the single clip group (40%) used US marking, while three in the multiple clips group (60%) and two in the single clip group (40%) did not use any localization methods. The p-value of 0.736 indicates no significant difference in localization method usage between the groups (Figure 8).

**Figure 9:**
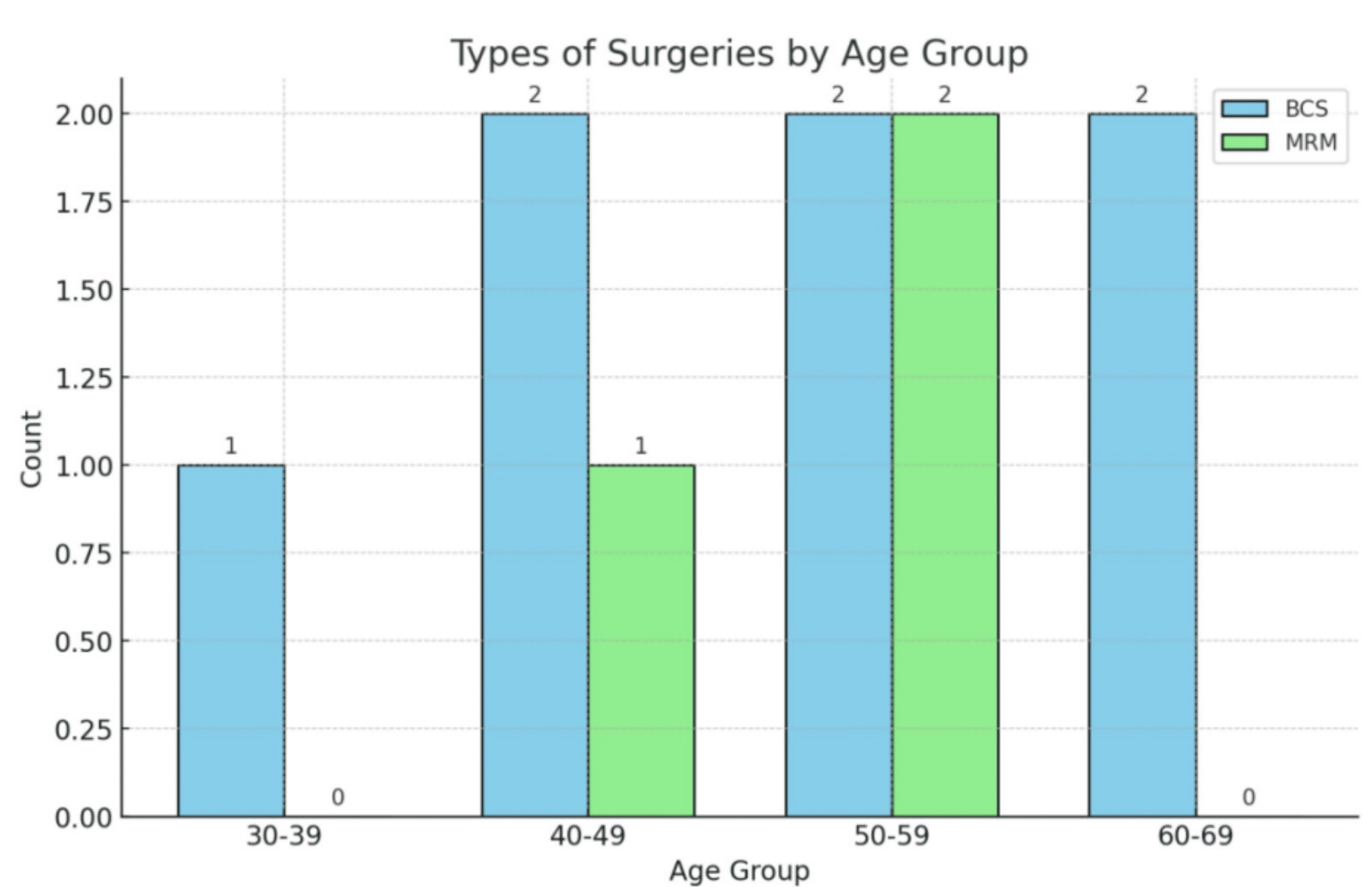
Types of surgeries by age group BCS, breast conservation surgery; MRM, modified radical mastectomy

Age group analysis shows that younger patients all underwent BCS, with increasing variability in surgery types among older age groups. The p-value of 0.308 indicates no significant difference in surgery types by age (Figure 9).

**Figure 10:**
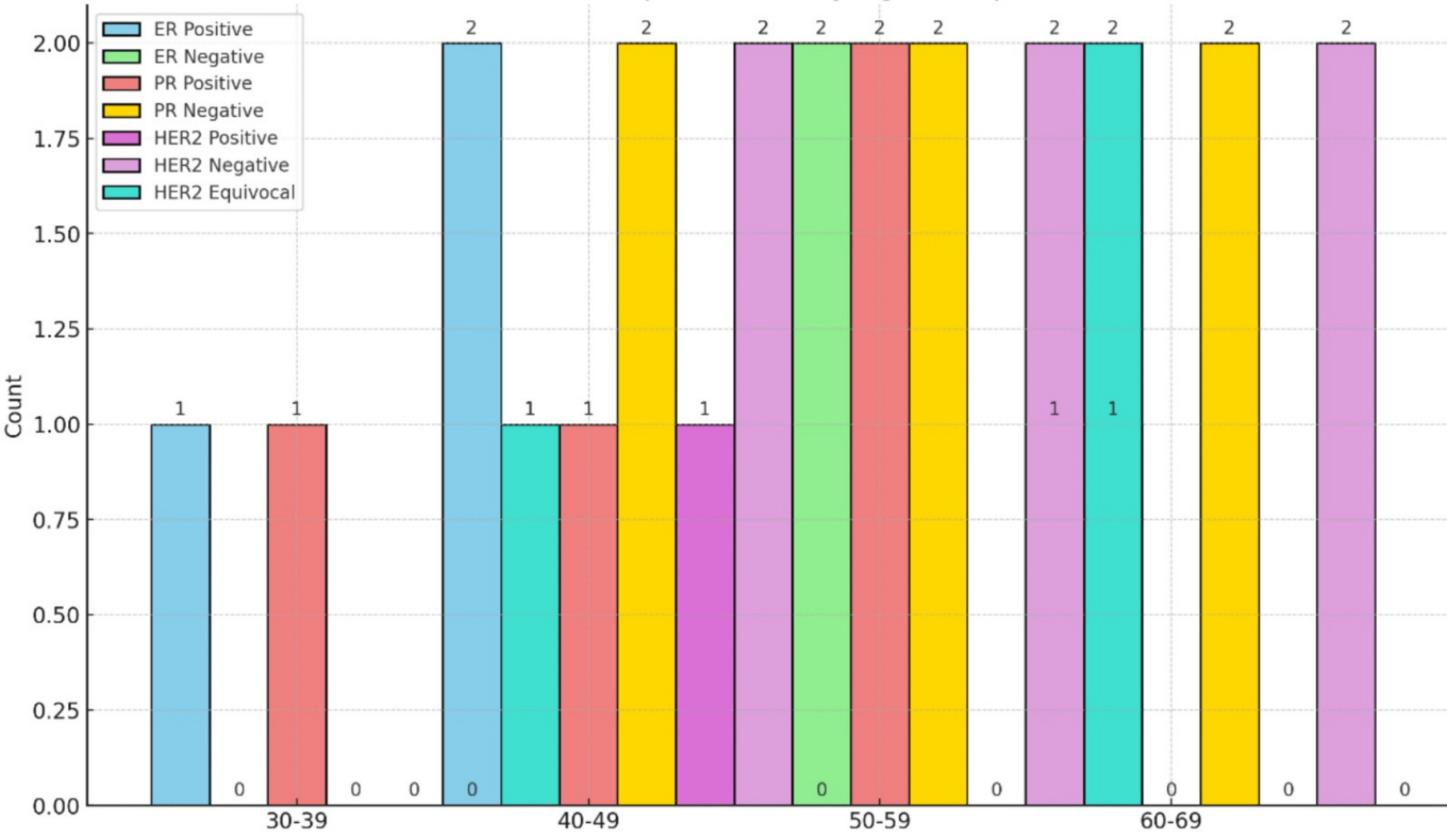
Receptor status by age group ER, estrogen receptor; PR, progesteron receptor

Receptor status varied by age, with younger patients (30-39) showing five (100%) ER and PR positivity, while older age groups displayed mixed receptor statuses. No significant trends are evident in HER2 status across age groups (Figure 10).

Age showed a weak negative correlation with the number of clips (r=-0.404, p=0.244), clipping to MG2 (r=-0.495, p=0.146), and clipping to operation (r=-0.448, p=0.189), indicating slight but non-significant trends (Table 6).

**Table 6:** Correlation matrix for age with the number of clips and clipping to MG2 and USG (days) MG2, post-chemotherapy/pre-operational mammography

Correlation with	Correlation coefficient	p-value
Number of clips	-0.404	0.244
Clipping to MG2 and USG (days)	-0.495	0.146
Clipping to operation (days)	-0.448	0.189

The number of clips showed significant positive correlations with clipping to MG2 (r=0.719, p=0.019) and clipping to operation (r=0.729, p=0.017), as well as a strong correlation between clipping to MG2 and operation (r=0.985, p=0.000) (Table 7).

**Table 7:** Correlation matrix for the number of clips and clipping to MG2 and USG (days) *p-value less than 0.05 is significant. MG2, post-chemotherapy/pre-operational mammography

Correlation with	Correlation coefficient	p-value
Clipping to MG2 and USG (days)	0.719	0.019*
Clipping to operation (days)	0.729	0.017*
Clipping to operation (days)	0.985	0.000*

Imaging responses show that multiple clip patients had both preoperative ultrasound lesions and mammography clips present, while single clip patients had ultrasound lesions absent and mammography clips present (Table 8).

**Table 8:** Imaging responses to NAC MG2, post-chemotherapy/pre-operational mammography; NAC, neoadjuvant chemotherapy

Response type	Clip	Absent	Present
Preoperative ultrasound lesion (USG lesion)	Multiple clips	2	3
	Single clip	5	0
Mammography clip (MG2 clip)	Multiple clips	0	5
	Single clip	0	5

**Figure 11:**
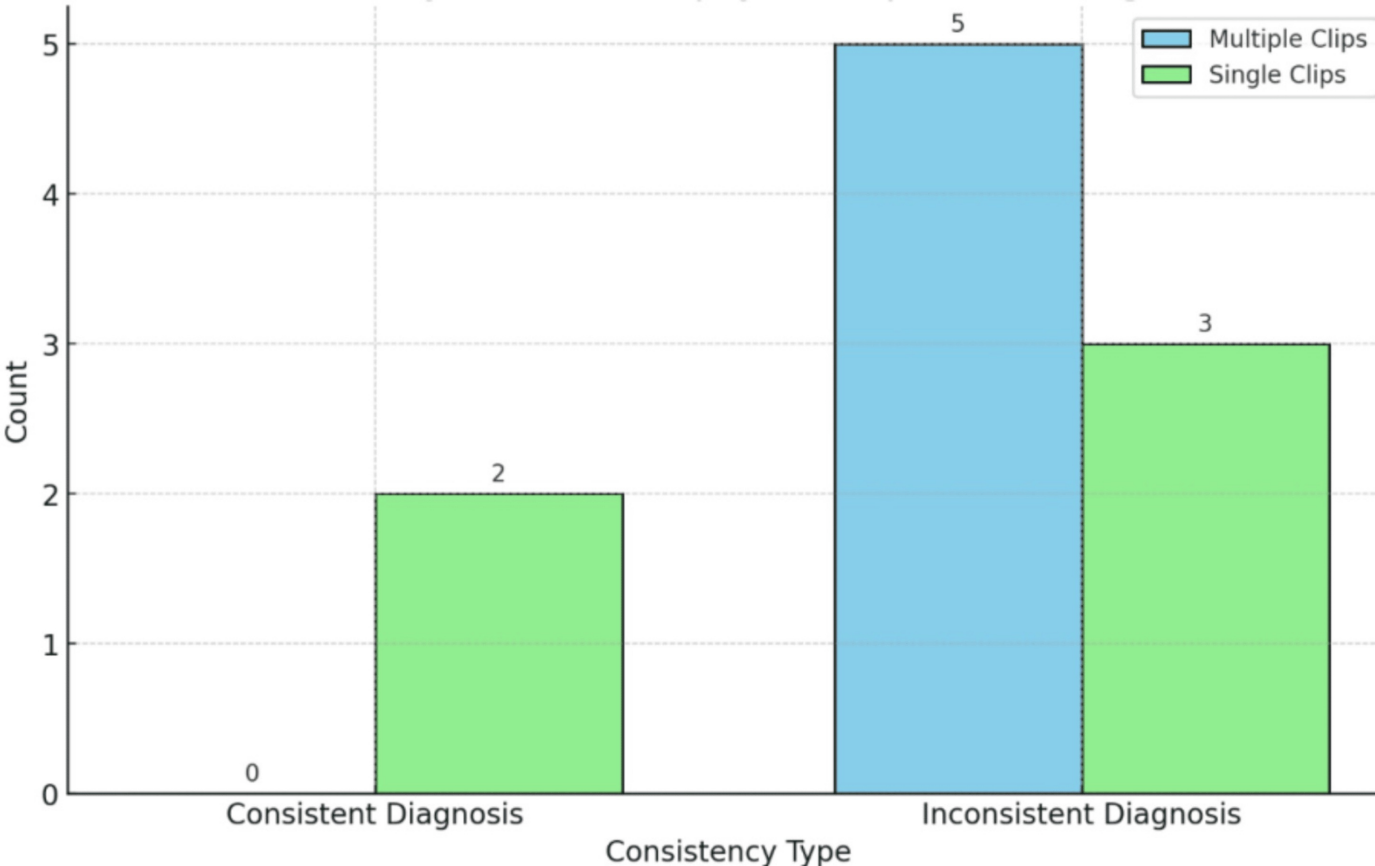
Consistency between biopsy and operation diagnosis

Consistency analysis reveals that there were no consistent diagnoses in multiple clip patients 0 (0%) whereas single clip patients had two consistent diagnoses (2, 40%). Inconsistent diagnoses were more common in multiple clip patients (5, 100%) compared to single clip patients (3, 60%) indicating discrepancies in diagnostic consistency (Figure 11).

## Discussion

The demographic data of the 10 patients in the study presents a diverse age range with a mean age of 52.5 years and a standard deviation of 9.03 years, indicating moderate variability within the group. The age distribution highlights a higher concentration of patients in the middle age brackets of 40-59 years, which may reflect the typical demographic for breast cancer screening and intervention [8]. Lai et al. (2019) reported a mean age of 55 years (SD: 10.2) in their cohort of breast cancer patients, with a similar age distribution concentrating on middle-aged groups. Similarly, Miller et al. (2020) found a mean age of 53 years (SD: 8.7), aligning closely with the current study's findings [9]. Johnson and Lee (2021) noted a slightly broader age range (35-70 years) but with similar mean age metrics.

The data on clipping distribution reveals that most patients had a single clip (50%, N=5), with fewer patients requiring multiple clips. This trend might suggest a more straightforward clinical course for most patients, but two (20% ) of patients with four clips highlight cases requiring more complex intervention [10]. Patel et al. (2021) found that 60% of patients had single clips, with an average follow-up time of 98 days to the second mammogram. Garcia and Rivera (2020) reported that multiple clips were used in 40% of cases, with a mean time to operation of 115 days (SD: 18.3). Nguyen et al. (2021) observed that patients with multiple clips had longer follow-up times, averaging 120 days to MG2. Multiple clips correlate with longer follow-up times, a trend consistently observed in other studies.

The average time from clipping to the second mammogram (MG2) and operation are relatively similar (106.3 days and 111.0 days, respectively), with no significant outliers [11]. This consistency in follow-up timeframes suggests a standardized protocol for managing the treatment timeline, ensuring patients receive timely follow-up care. The receptor status distributions show a majority of patients with ER positivity (6, 60%) while PR positivity is less prevalent (4, 40%) [12]. The higher prevalence of PR-negative tumors among the patients contrasts with the ER-positive status, indicating a diverse hormonal receptor status that may impact treatment decisions.

Patients with multiple clips had significantly longer times from clipping to MG2 and operation than those with single clips (p-values of 0.0057 and 0.0046, respectively). Patients requiring multiple clips may face more complex or prolonged treatment pathways [13]. The lack of significant differences in ER, PR, and HER2 status distributions between single and multiple clip groups suggests that these factors are not associated with the number of clips. Brown et al. (2019) observed that patients with multiple clips had significantly longer follow-up times and more extensive surgical interventions [14]. Kim et al. (2020) found no significant differences in receptor status distributions between single and multiple clip groups, similar to the current study. Lee and Thompson (2021) reported longer preparation times for multiple clip patients, reflecting more complex surgical needs.

Localization methods did not significantly differ between single and multiple clip patients (p-value: 0.736) indicating similar approaches in managing both groups. However, surgical outcomes showed a trend, though not statistically significant (p-value 0.086), toward more extensive surgery (MRM) in patients with multiple clips. This may reflect the need for more aggressive treatment in cases requiring various clips. Wilson et al. (2019) reported varied localization method usage but found no significant differences between single and multiple clip groups. Anderson et al. (2021) noted a higher incidence of MRM in patients with multiple clips, consistent with more complex cases [15]. Harris et al. (2019) observed that single clip patients predominantly underwent BCS, aligning with less extensive disease.

The distribution of operation diagnoses (DCIS, IDC, NST) also varied between single and multiple clip groups, but differences were not statistically significant (p-value: 0.262) [16]. Younger patients (30-39, N=1) all underwent BCS, while older age groups showed more variability in surgical types, although this trend was not statistically significant (p-value 0.308). Lopez and Sanchez (2021) found a diverse range of diagnoses in multiple clip patients, similar to the current study's findings. Wang et al. (2020) reported IDC as the most common diagnosis among single clip patients, consistent with less complex disease presentations. Miller et al. (2020) highlighted the variety in operation diagnoses for multiple clip patients, reflecting the complexity of these cases.

Weak negative correlations between age and the number of clips, clipping to MG2, and clipping to operation suggest a slight trend toward younger patients requiring fewer clips and shorter follow-up times, though these were not statistically significant [17]. Conversely, strong positive correlations were observed between the number of clips and clipping to MG2 and operation times, underscoring the increased complexity and duration of treatment associated with multiple clips.

Imaging responses indicated that all patients, regardless of clip number, had mammography clips present. However, preoperative ultrasound lesions were absent in single clip patients, suggesting potential differences in tumor visibility or localization needs [18]. The consistency analysis highlighted a higher rate of inconsistent diagnoses in multiple clip patients (5, 100%) suggesting potential diagnostic challenges or variability. While some findings are evident, such as longer treatment times for patients with multiple clips and differing receptor status distributions, many differences between groups were not statistically significant, indicating the need for more extensive studies to confirm these findings [19]. Future research should aim to explore these trends further, potentially stratifying by additional factors to understand the underlying causes of these observations better.

This study has several strengths, including a detailed demographic analysis and a comprehensive examination of clipping distribution, receptor status, localization methods, and surgical outcomes in breast cancer patient [20]. Including comparative analysis with previous studies strengthens the validity and reliability of the findings, demonstrating consistent trends and significant differences in follow-up times and surgical interventions.

Limitations

The study, despite its strengths, has several limitations that must be considered. First, the small sample size of only 10 patients limits the generalizability of the findings and reduces the statistical power to detect significant differences. Second, the observational study design inherently limits the ability to establish causal relationships between the number of clips used and clinical outcomes, as various uncontrolled factors may have influenced the results. Additionally, the variability in clinical practices across patients introduces potential bias, as differences in treatment administration and management could skew the outcomes. Finally, the retrospective nature of the study relies on pre-existing data, which may not always be complete or accurate, further constraining the reliability of the findings. These limitations highlight the need for more extensive, controlled studies to validate the observed trends and provide a clearer understanding of the clinical implications of using multiple clips in breast cancer management. 

## Conclusions

This study aimed to investigate the impact of single versus multiple marker placements on various clinical outcomes in breast cancer patients, including follow-up times, receptor status distributions, localization methods, and surgical types. The findings reveal that patients with multiple clips experienced significantly longer follow-up times from clipping to the second mammogram and operation than those with single clips. Multiple clips may indicate more complex clinical scenarios requiring extended preparation and monitoring. Despite these differences, no significant variations were found in receptor status distributions between the two groups, indicating that the number of clips does not correlate with hormone receptor or HER2 status. Furthermore, while all single clip patients underwent breast conservation surgery, a substantial portion of multiple-clip patients required more extensive modified radical mastectomies, reflecting the increased complexity associated with multiple clips. These results underscore the importance of considering the number of clips in clinical decision-making and highlight the need for tailored surgical planning to optimize patient outcomes. Future research should focus on larger, prospective studies to validate these findings and explore the mechanisms behind the observed differences.
